# Dictators Differ From Democratically Elected Leaders in Facial Warmth

**DOI:** 10.1177/1948550621991368

**Published:** 2021-02-04

**Authors:** Miranda Giacomin, Alexander Mulligan, Nicholas O. Rule

**Affiliations:** 1 Department of Psychology, 3151 MacEwan University, Edmonton, Alberta, Canada; 2 7938 University of Toronto, Toronto, Ontario, Canada

**Keywords:** face perception, leadership, dictators, democrats

## Abstract

Despite the many important considerations relevant to selecting a leader, facial appearance carries surprising sway. Following numerous studies documenting the role of facial appearance in government elections, we investigated differences in perceptions of dictators versus democratically elected leaders. Participants in Study 1 successfully classified pictures of 160 world leaders as democrats or dictators significantly better than chance. Probing what distinguished them, separate participants rated the affect, attractiveness, competence, dominance, facial maturity, likability, and trustworthiness of the leaders’ faces in Study 2. Relating these perceptions to the categorizations made by participants in Study 1 showed that democratically elected leaders looked significantly more attractive and warmer (an average of likability and trustworthiness) than dictators did. Leaders’ facial appearance could therefore contribute to their success within their respective political systems.

People make intuitive judgments about others when deciding who should lead and whom they will follow. Drawing on their implicit theories of leadership, perceivers approach leader selection with a set of expectations relative to the values that they personally hold for what matters in a leader (e.g., Berger & Wagner, 2007; Lord & Maher, 1991; Spisak et al., 2012). Yet they also show notable consensus about the attributes that make a leader effective (Penton-Voak et al., 2006; Rule et al., 2008, 2010), including both behavioral and physical attributes (Giacomin & Rule, 2020; Olivola, Funk, & Todorov, 2014; Todorov et al., 2015). People therefore show a bias to select leaders that look powerful or warm, depending on the context in which the leader will need to act (e.g., Little, 2014; Olivola & Todorov, 2010a; Re & Rule, 2016; Rule et al., 2010). A portion of this evaluation stems from inferences about leadership candidates’ facial appearance.

The face hosts cues to attributes that support more global impressions of a person’s social status, personality traits, and (most pertinent) political affiliation (Alaei & Rule, 2016; Sutherland et al., 2015; Zebrowitz & Montepare, 2008). Impressions of others can show moderate levels of accuracy based on minimal amounts of facial information (Re & Rule, 2015; Tskhay & Rule, 2013). Even in cases for which perceivers demonstrate biases that cause them to extract this information inaccurately from faces (or in which a criterion for accuracy does not exist; Hassin & Trope, 2000; Olivola & Todorov, 2010b; Stoker et al., 2016; Todorov et al., 2015), their consensual impressions seem to influence their decisions and actions (Olivola, Funk, & Todorov, 2014; Todorov et al., 2015). For instance, both adults and children can pick the winner of foreign elections based on quick judgments of politicians’ facial photographs, suggesting that their opinions about who looks like a better leader match the ostensibly better-informed decisions of the voters in the actual election (Antonakis & Dalgas, 2009).

Leaders’ faces may influence their electoral success but may also predict the political system in which they govern. Although far from perfect (Benjamin & Shapiro, 2009; Olivola & Todorov, 2010b; Saxton et al., 2019), people differentiate between Republican and Democratic U.S. Senate candidates based on their faces (Carpinella & Johnson, 2013; Jahoda, 1954; Laustsen & Petersen, 2016; Olivola et al., 2012; Rule & Ambady, 2010a; Wilson & Rule, 2014) and can reliably infer the ideology of Swiss parliament members, as reflected by their voting records (Samochowiec et al., 2010). Subtle cues seem to drive these judgments (e.g., Peterson et al., 2018). For example, perceivers generally expect Republicans to look more powerful, which they actually do (Rule & Ambady, 2010a; but see also Wilson & Rule, 2014). Ballew and Todorov (2007) found that participants rated the faces of the winners of the 2006 U.S. gubernatorial elections as more competent. Indeed, voters’ impressions of political candidates’ attributes from facial appearance influence how they vote in democratic elections across the globe (e.g., Giacomin & Rule, 2020; Lawson et al., 2010; Little et al., 2007).

Notably, these judgments fluctuate depending on contextual factors such as the political or organizational climate. For example, people favor leaders with dominant and attractive faces during war times but with more feminine and trustworthy faces when at peace (e.g., Little et al., 2012) and prefer dominant-looking individuals when selecting team members for intergroup competitions but warm-looking individuals in cooperative settings (e.g., Hehman et al., 2015; Little, 2014; Little et al., 2007; Re et al., 2013). Different types of organizations also appear to select specific leaders: CEOs of nonprofit organizations look warmer and less dominant than CEOs of for-profit organizations, and the leaders of mafia families express different social traits than the leaders of law firms (Re & Rule, 2016, 2017). Leaders’ facial appearance may also be domain-specific: People could accurately distinguish military (i.e., Army Generals) and sport leaders (i.e., coaches) from business (i.e., CEOs), but not political (i.e., Governors), leaders because sport and military leaders look less attractive and colder than business leaders (Olivola, Eubanks, & Lovelace, 2014). Leaders may therefore share common facial features or characteristics within a domain. Contextual factors surrounding a leader’s election thus influence the attributes that constituents and voters value (e.g., political affiliation, current political climate, domain of leadership; Little et al., 2012; Olivola, Eubanks, & Lovelace, 2014; Olivola, Funk, & Todorov, 2014).

Although past research has examined how geographic, economic, and organizational context can influence perceptions of political leaders’ faces, most research has examined democratic political systems, which rely on constituents to select leaders though majority rule voting. Researchers have yet to examine leaders from authoritarian regimes, which do not typically extend voting rights to the general population but allow a single absolute ruler to govern the society (Bobbio, 1989). Because authoritarian leaders, sometimes referred to as dictators, characteristically employ oppressive practices to control threats to their power, they may appear quite dominant and aggressive compared to their freely elected counterparts. We thus investigated how leaders’ perceived attributes may vary between different political systems by focusing on the attributes inferred from authoritarian and democratically elected politicians’ faces.

## The Current Research

The existing research relating perceptions of politicians’ attributes and party membership to their professional outcomes has primarily focused on democratically or freely elected leaders. But roughly 61% of the world’s population live outside of a free and democratic society (Abramowitz, 2018). Here, we examine perceptions of dictators’ traits, which could help to develop an understanding of how they attain and maintain power. In Study 1, we tested whether participants could classify photos of past and present heads of state according to their leadership style (i.e., as dictators or democrats). Here, we use the term “democrat” for leaders freely elected by their constituents (regardless of their political values). Based on previous research that found people could differentiate politicians based on their political orientation (e.g., Carpinella & Johnson, 2013), we hypothesized that people would be able to accurately distinguish democrats from dictators. In Study 2, we examined the attributes that participants infer from the leaders’ photos to support these classifications. Past research suggests that the face fits the leader context (Olivola, Eubanks, & Lovelace, 2014). Here, we hypothesized that leaders will be perceived as equally powerful due to their high-status positions but that perceptions of facial warmth will distinguish democrats from dictators. Dictators are expected to appear cold and unfriendly, whereas democrats may be expected to appear warm and trustworthy. To help leaders maintain power, their faces may need to appear congruent with the value of their political system. Together, these studies go beyond previous research by examining differences between real democrats and dictators from across the world and further extend theorizing about the context-dependent nature of leader face perception.

## Study 1

Past research has demonstrated that leaders with opposing ideologies within a single type of political system look different in facial photographs, allowing perceivers to categorize them according to their political party with moderate accuracy (e.g., Rule & Ambady, 2010a; Samochowiec et al., 2010; though also see Olivola & Todorov, 2010b; Saxton et al., 2019). Here, we hypothesized that opposing ideologies *between* political systems might similarly allow perceivers to categorize leaders according to their particular leadership style. We presented participants with the photos of past and present democratic (freely elected) and dictatorial (authoritarian) heads of state and asked them to classify each person as a democrat or dictator.

## Method

### Materials

We cross-referenced two internationally recognized rating systems of state freedom to generate a list of candidate dictators and democrats: The Economist Intelligence Unit’s *Democracy Index* and the Freedom House’s *Freedom of the World* report (Puddington & Roylance, 2017; The Economist, 2016). The *Democracy Index* rates countries from “democratic” to “authoritarian” based on human rights, electoral process, government functioning, and political culture. The *Freedom of the World* report rates countries from “free” to “not free” based on electoral process, multiparty politics, and public access to politics. We defined dictators as leaders from countries rated as “authoritarian” or “not free” and democrats as freely elected leaders from countries ranked as “free” or “democracies.”

In the interest of statistical power, we included past leaders.^1^ To select former dictators, we focused on countries that lacked open elections, had a known history of human rights abuses, or had leaders who came to power via military coups. Conversely, we selected former democratic leaders from countries with a history of free and open elections and no human rights abuses. This yielded 160 male heads of state: 80 democratic leaders (31 still in power, 49 no longer in power; 50% Caucasian, 25% Black, 12.5% Hispanic, 6.3% East Asian, and 6.3% South Asian) and 80 dictators (34 still in power, 46 no longer in power; 32.5% Black, 26.3% Arab, 12.5% Caucasian, 13.8% East Asian, 11.3% Hispanic, 2.5% South Asian, and 1% unknown).

We chose photos in which the leaders looked at the camera and expressed limited emotion. We grayscaled and cropped the photos just below the chin, at the crown of the head, and as close to the ears as possible, including any head coverings (to allow the most realistic picture possible). We excluded well-known heads of state to minimize recognition (e.g., Vladimir Putin, Justin Trudeau, Donald Trump) and female heads of state to avoid gender biases in participants’ ratings (Rule & Ambady, 2009).

### Participants

Results of a power analysis indicated that 70 participants would provide 95% power to obtain an effect size equal to that found in Rule and Ambady’s (2010b) study on the classification of U.S. Senate candidates (*d* = 0.44) with a 5% false-positive rate for a two-tailed one-sample *t* test. We oversampled to guard against attrition, recruiting 90 American Mechanical Turk (MTurk) workers (*N* = 90; 41 men, 49 women; 72% Caucasian; *M*_age_ = 34.94 years, *SD* = 10.80).

### Procedure

Participants read that they would see photographs of several different people, classifying each as a dictator or democrat. They viewed 160 faces individually in random order, categorizing them at their own pace. Participants did not know the proportion of democrats and dictators included. Apart from demographic information, we included no other measures or manipulations and did not exclude any participants.^2^ At the end of the study, participants listed the names of leaders that they recalled so that we could exclude those responses (0.01% of all trials).

## Results

We analyzed the data using signal detection statistics, wherein correct categorizations of democrats as democrats constituted hits (*M* = .71, *SD* = 0.17) and incorrect categorizations of dictators as democrats constituted false alarms (*M* = .51, *SD* = 0.18), using these values to calculate detection (*A*′) and response bias (*B″*) values for each participant (Macmillan & Creelman, 2004). Participants categorized targets as democrats significantly more often than as dictators (*B″*: *M* = −.18, *SD* = 0.29), *t*(89) = −37.93, *p* < .001, *d* = 2.84. Despite this bias, they nevertheless categorized the leaders significantly better than chance (*A*′: *M* = .69, *SD* = 0.09), *t*(89) = 70.84, *p* < .001, *d* = 7.64.

## Discussion

Past research has examined whether people could identify individuals’ ideology within a single type of political system (democracy; e.g., Olivola & Todorov, 2010b; Rule & Ambady, 2010a). Here, we extended this to ideologies between *different* types of political systems. The average participant correctly distinguished unfamiliar democrats’ and dictators’ leadership style about 69% of the time from facial photographs. Participants’ biased response (i.e., choosing democrat more often than dictator) may reflect that individuals living in a democratic society tend to overestimate the number of democratically elected leaders in the world (see Olivola & Todorov, 2010b). We proceeded to investigate the basis for these categorizations in Study 2, hypothesizing that more dominant faces serve dictators better, whereas trustworthy faces might fit more with proponents of democracy.

## Study 2

Past research relating freely elected leaders’ electoral success and party membership to perceptions of their faces has largely focused on affect, attractiveness, competence, dominance, facial maturity, likability, and trustworthiness (Giacomin & Rule, 2020). We therefore asked participants in Study 2 to evaluate the leaders from Study 1 along these same dimensions to explore how people distinguish democrats from dictators. We applied a lens model analysis (Brunswik, 1952) to determine which traits predicted the leaders’ group memberships (i.e., valid cues) and which traits predicted participants’ consensus perceptions of the leaders in Study 1 (i.e., utilized cues). Given that contextual factors may influence the attributes that constituents value, we hypothesized that world leaders from different political systems may differ in terms of how warm they appear (e.g., Re & Rule, 2017).

## Method

### Materials

We used the same 160 photos from Study 1 (80 dictators and 80 democrats).

### Participants

We predetermined our sample size to 30 perceivers per judgment, which should allow for stable mean impressions of each target (Hehman et al., 2018), totaling 229 MTurk workers after excluding 41 participants who responded uniformly (*n* = 36) or suggested that we ought not to use their data (*n* = 5).

Each participant rated all photographs on a single attribute in a between-subjects design: attractiveness (*N* = 23; 14 men, nine women; 56% Caucasian; *M*_age_ = 32.30 years, *SD* = 11.59; α = .99), babyfacedness (*N* = 26; six men, 20 women; 88% Caucasian; *M*_age_ = 33.19 years, *SD* = 8.59; α = .99), competence (*N* = 25; 10 men, 15 women; 80% Caucasian; *M*_age_ = 35.36 years, *SD* = 11.89; α = .99), dominance (*N* = 27; nine men, 18 women; 70% Caucasian; *M*_age_ = 37.59 years, *SD* = 10.40; α = .99), likability (*N* = 25; 10 men, 15 women; 80% Caucasian; *M*_age_ = 32.67 years, *SD* = 8.82; α = .97), negative affect (i.e., sadness; *N* = 30; 12 men, 18 women; 76% Caucasian; *M*_age_ = 34.16 years, *SD* = 11.18; α = .99), photo quality (*N* = 23; 11 men, 12 women; 87% Caucasian; *M*_age_ = 33.82 years *SD* = 10.98; α = .98), positive affect (i.e., happiness; *N* = 24; 14 men, 10 women; 67% Caucasian; *M*_age_ = 31.21 years, *SD* = 9.57; α = .98), and trustworthiness (*N* = 26; seven men, 18 women, and one other; 81% Caucasian; *M*_age_ = 31.73 years, *SD* = 7.19; α = .98).

### Procedure

Participants rated each face along one dimension from 1 (*not at all*) to 8 (*extremely*) in response to the question “How *X*?” For photo quality, participants rated the quality of the photos from 1 (*very poor*) to 8 (*excellent*). For positive and negative affect, we respectively asked “How happy?” from 1 (*very unhappy*) to 8 (*very happy*) and “How sad?” from 1 (*not at all sad*) to 8 (*very sad*), reverse coding the latter and averaging the two scores into a composite affect judgment (α = .96). At the end of the study, participants again indicated whether they recalled any of the faces and provided demographic information; we report all measures, manipulations, and exclusions. In addition, the second author coded the photographs of each leader for whether he had facial hair (0 = no facial hair, 1 = facial hair), wore glasses (0 = no glasses, 1 = glasses), and had prior military experience (0 = no experience, 1 = prior experience). Power analysis indicated that we had more than 99% power to conduct two-tailed, two-sample *t* tests in which the targets constituted the unit of analysis based on the effect size obtained in Study 1.

## Results

We averaged participants’ ratings across leaders to compute a single score for each leader’s affect, attractiveness, competence, dominance, facial maturity (i.e., reverse-coded babyface ratings), likability, photo quality, and trustworthiness. Facial hair, presence of glasses, and photo quality served as control variables because they can influence social perceptions (Rule et al., 2017). Olivola, Eubanks, and Lovelace (2014) found that people had difficulty distinguishing between military and political leaders, perhaps due to overlapping career paths. Because dictators are often former military leaders and democratically elected leaders are often career politicians or business leaders, we statistically adjusted for prior military experience in the lens model analysis. We likewise kept affect, attractiveness, and competence^3^ separate because they play a role in leadership perceptions (e.g., Banducci et al., 2008; Berggren et al., 2010). We created two composites for each leader with the remaining judgments: warmth (likability and trustworthiness; α = .87) and power (dominance and facial maturity; α = .57).

### Mean-Level Differences

We began by investigating differences in the perceptual judgments between dictators and democrats. Comparisons of the mean ratings given to the democrats and dictators showed that the democrats looked warmer, *t*(158) = 4.20, *p* < .001, *d* = 0.67, more attractive, *t*(158) = 4.78, *p* < .001, *d* = 0.76, more competent, *t*(158) = 4.06, *p* < .001, *d* = 0.65, happier, *t*(158) = 4.20, *p* < .001, *d* = 0.67, had higher quality photographs, *t*(158) = 4.01, *p* < .001, *d* = 0.64, less often donned facial hair, χ2(1, *N* = 160) = 10.80, *p* = .002, Cramer’s ϕ = .01, and had less prior military experience, χ2(1, *N* = 160) = 45.14, *p* < .001, Cramer’s ϕ = .01, than dictators (Table 1). Dictators looked marginally more powerful than democrats, *t*(158) = −1.75, *p* = .08, *d* = 0.28. The types of leaders did not differ in whether they wore glasses, χ^2^(1, *N* = 160) = 1.72, *p* = .26, Cramer’s ϕ = .01.

**Table 1.** Descriptive Statistics for the Judgments in Study 2 by Leader Type.

Judgment	Democrats		Dictators	
	*M*	*SD*	*M*	*SD*
Affect*	4.91	.92	4.29	.92
Attractiveness*	3.56	.61	3.13	.52
Competence*	5.16	.44	4.86	.50
Power	4.74	.64	4.92	.63
Warmth*	4.42	.59	4.01	.66
Control variables				
Photo quality*	5.00	.55	4.65	.58
Facial hair* (%)	14		36	
Glasses (%)	28		19	
Military experience* (%)	11		63	

* Leader types significantly differ at *p* < .05.

Using the data from Study 1, we calculated the proportion of instances in which participants categorized each target as a democratic leader and correlated that consensus perception with the mean rating for each trait (Bonferroni corrected α = .001). Participants in Study 1 tended to perceive the targets as democrats when they appeared warmer (*r* = .70, *p* < .001), less powerful (*r* = −.35, *p* < .001), more attractive (*r* = .54, *p* < .001), competent (*r* = .52, *p* < .001), happier (*r* = .51, *p* < .001), had higher quality photographs (*r* = .36, *p* < .001), less facial hair (*r* = −.25, *p* < .001), and no military experience (*r* = −.43, *p* < .001). The presence of glasses did not relate to perceived leadership style (*r* = .02, *p* = .76); we therefore did not include it in the subsequent lens model analysis.^4^

### Lens Model Analysis

A multiple mediation–based lens model analysis (Back et al., 2010) using Hayes’s (2013) PROCESS code with 5,000 bootstraps helped to determine which judgments (i.e., affect, attractiveness, competence, power, and warmth) related to actual leadership style (coded: 0 = dictator, 1= democrat) and perceived leadership style (based on the consensus proportions from Study 1). We included the presence of facial hair, photo quality, and prior military experience as control variables, though the results remained the same when excluding them.

The total effect of actual leadership style on perceived leadership style was significant, *b* = .13, *SE* = .03, 95% confidence intervals (CI) [.07, .19], *t*(155) = 4.12, *p* < .001, when statistically adjusting for facial hair, photo quality, and prior military experience. The direct effect was reduced, though remained significant, *b* = .07, *SE* = .02, 95% CI [.03, .12], *t*(150) = 3.03, *p* = .003, after simultaneously including each of our dependent variables and statistically adjusting for control variables. Thus, people’s inferred leadership style from leaders’ faces accurately predicted leaders’ actual leadership style when controlling for important variables related to face perception (e.g., facial hair, prior military experience).

**Figure 1.**
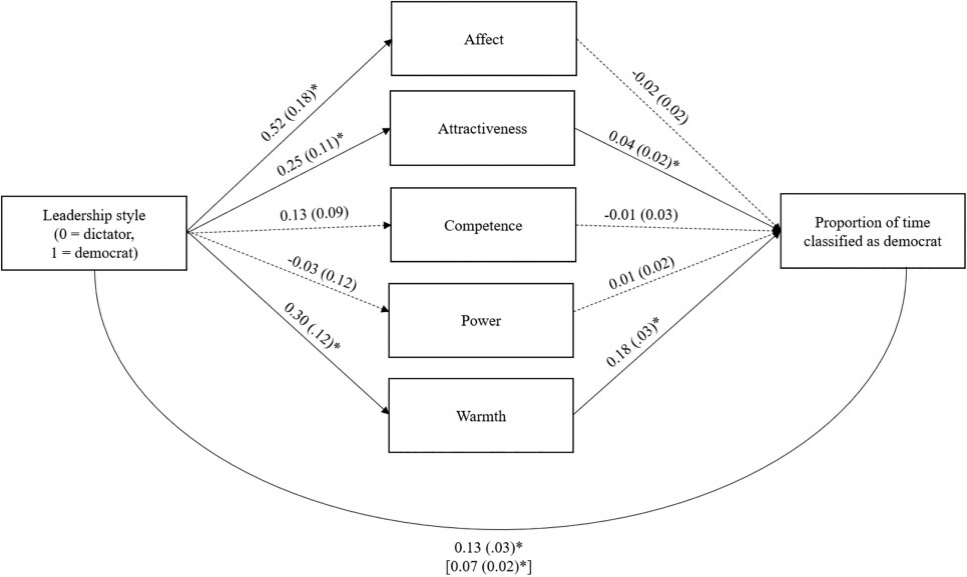
Unstandardized coefficients (standard errors in parentheses) from the multiple mediation–based lens model analysis testing how perceptions of affect, attractiveness, competence, power, and warmth explain the association between actual and perceived leadership style (controlling for facial hair, photo quality, and prior military experience). *Note.* Solid and dotted lines indicate significant and nonsignificant paths, respectively. *p < .05.

In terms of utilized cues, attractiveness, *b* = .04, *SE* = .02, 95% CI [.005, .08], *t*(150) = 2.21, *p* = .03, and warmth, *b* = .18, *SE* = .03, 95% CI [.12, .24], *t*(150) = 6.19, *p* < .001, predicted perceived leadership style, but affect, *b* = −.02, *SE* = .02, 95% CI [−.05, .02], *t*(150) = −1.01, *p* = .32, competence, *b* = −.01, *SE* = .03, 95% CI [−.07, .04], *t*(150) = −0.46, *p* = .65, and power did not, *b* = −.01, *SE* = .02, 95% CI [−.03, .05], *t*(150) = 0.36, *p* = .72 (Figure 1). In terms of valid cues, affect, *b* = .54, *SE* = .18, 95% CI [.17, .90], *t*(155) = 2.91, *p* = .004, attractiveness, *b* = .25, *SE* = .11, 95% CI [.04, .46], *t*(155) = 2.38, *p* = .02, and warmth, *b* = .30, *SE* = .12, 95% CI [.06, .54], *t*(155) = 2.45, *p* = .02, but not competence, *b* = .13, *SE* = .09, 95% CI [−.04, .30], *t*(155) = 1.47, *p* = .14, or power, *b* = −.03, *SE* = .12, 95% CI [−.27, .22], *t*(155) = −0.23, *p* = .82, predicted actual leadership style.

Notably, tests of the indirect effects demonstrated that attractiveness, *b* = .01, *SE* = .01, 95% CI [.01, .03], and warmth, *b* = .05, *SE* = .03, 95% CI [.003, .11], mediated the association between actual and perceived leadership style, whereas affect, *b* = −.01, *SE* = .01, 95% CI [−.03, .01], competence, *b* = −.002, *SE* = .01, 95% CI [−.01, .01], and power did not, *b* = −.0002, *SE* = .003, 95% CI [−.006, .01]. Thus, attractiveness and warmth may be the primary characteristics supporting the detection of leadership style, explaining how participants arrived at their categorizations of the leaders as dictators versus democrats.

## Discussion

In Study 2, we investigated the cues that relate to actual leadership style (cue validity) and perceptions of leadership style (cue utilization). The lens model suggests that people expect warmer and more attractive leaders to lead democratic nations and that the leaders of democratic nations do indeed look warmer and more attractive than dictators (even when statistically adjusting for facial hair, photo quality, and prior military experience). These traits seem sensible in a democratic context where popularity plays a critical role in whether someone emerges as a leader. In contrast, looking colder and less attractive might similarly facilitate the command of authority on which dictators rely to control the citizens of their nations. For instance, role congruity theory posits that people prove more effective when their perceived attributes match the expectations of their roles (Eagly & Diekman, 2005). Thus, not only might democratic leaders who look attractive and warm have a potential edge in popular elections, dictators who look the opposite (e.g., unattractive and cold) might hold advantages that allow them to maintain power in the face of threats.

## General Discussion

Freedom around the world has been decreasing: 112 countries have seen net declines in measures of freedom (Abramowitz, 2018). Despite these alarming trends, no research has investigated how people perceive dictators or how the ways that dictators visually present themselves might facilitate their reign. Here, we examined whether the faces of authoritarian and democratically elected leaders look systematically different. In Study 1, participants classified photos of politicians as dictators or democrats significantly better than chance. In Study 2, we found that traits related to warmth (trustworthiness and likability) and attractiveness, but not power (dominance and facial maturity) or competence, allow people to make this distinction even after statistically adjusting for a host of variables related to face perception (i.e., facial hair, photo quality, and prior military experience). Leaders’ appearances may therefore contribute to the contexts in which they reign.

This research extends previous work examining the context-dependent nature of perceptions of leaders by focusing on the type of leadership style. Indeed, we find that the face suits the leadership style. Democracies value justice, openness, and transparency; as such, voters prefer politicians whose faces convey warmth through trustworthiness and likability. Dictatorships, however, operate through subordination of the population, deception, and tyranny. Dictators who appear harsh and less warm match this style of governance better and might therefore successfully elicit more fear and intimidation in the population. In contrast, a dictator who looks warm may not be taken as seriously as a harsh-looking dictator when that person attempts to subjugate the population. Democratically elected leaders who appear warm may thus be more likely to attain and maintain power if their faces appear congruent with that value of their political system.

### Limitations

The binary classification of leadership style limits the current research. Countries vary in freedom, with some democracies freer than others and some dictatorships more constrained. Indeed, measures of freedom and democracy approximate dictatorships in many democratic countries. Furthermore, many countries elect leaders who begin to reverse gains made in freedom and democracy while still maintaining democratic status (Abromowitz, 2018). Participants’ binary classifications thus fail to capture this nuance.

Likewise, using photographs of real-world leaders improves the external validity of our findings but also introduces noise. Although we accounted for some potential covariates (e.g., photo quality, facial hair, prior military experience), additional important factors may remain outstanding (e.g., race). Low reliability in the composite power measure may also partly explain why it did not predict perceptions of leadership style but warmth did. Despite this limitation, both democrats and dictators hold a great deal of power in governing entire countries. Just as past work has shown that the appearance of exceptional traits predicts leadership in the mafia (i.e., social skill) and law firms (i.e., power; Re & Rule, 2017), perceptions of warmth may primarily distinguish dictators and democratically elected leaders because it constitutes an unrequired, exceptional trait among heads of state (who all look powerful).

### Future Directions

Given their widespread power, it is important to understand how politicians are perceived, elected, and maintain their power. The current studies add to a growing body of research suggesting that the same face may not hold the same leadership value across all situations. Different leadership contexts may activate distinct leader prototypes (e.g., Olivola, Eubanks, & Lovelace, 2014). Here, we found that democratically elected leaders appear much more attractive and warmer than authoritarian leaders. Future research should continue to examine how context interacts with person perception to determine who is selected and successful as a leader.

Similarly, we recruited participants from the United States, a democratic society. As such, they may perceive leaders differently than people who live under a dictator’s rule. Future research should thus examine how people living in autocratic versus democratic societies perceive leaders in different ways. For example, perceptions of warmth may bias people’s judgments of politicians depending on their degree of civil rights. Specifically, warmer faces may elicit more forgiveness from the population for their transgressions, which may provide important information about how and why certain leaders remain in power, despite harming their nations.

In addition, research could examine whether some individuals are bound to be leaders of democratic or autocratic states. Research has found that inferences of leaders’ power from yearbook photos can predict corporate success, indicating stability in traits used to predict success (Rule & Ambady, 2011). It may be possible to predict leadership style from photos of politicians before they even entered positions of power (e.g., Rule & Ambady, 2010a). Doing so could indicate that dictators and democrats vary systematically in facial structure and that this facial structure exists across the life span, leading to different positions of power.

### Conclusion

People perceive differences between dictators and democrats based on facial photographs. Dictators and democrats vary in perceived attractiveness and warmth, and these cues guide people in categorizing politicians as dictators versus democratically elected leaders. A common question asked of authoritarian regimes is why people stand by such leaders. This research offers one possible explanation: Their facial appearance matches the political systems in which they operate. Democracies thrive on honesty and justice, so leaders with warmer faces may more easily get elected to power. Harsher, less warm faces match dictatorships, which run on domination and deception; so, appearing intimidating might help to inspire the fear needed to dominate a population. People may therefore readily follow those who suit their impressions of the political system more broadly.

## Notes

1. We conducted a series of additional analyses examining differences in perceptions of past and current autocratic and democratic leaders. There were minimal differences between current and past political leaders, and time of leadership did not interact with leadership style on any of the primary dependent variables. We therefore analyze the results collapsing across current and past leaders, though results remain the same when controlling for time of leadership.

2. Studies 1 (https://osf.io/mnst6/?view_only=aedce1b1113d4d2ea5dacd13811b75f7) and 2 (https://osf.io/r7vm4/view_only=7df8e05d89154a218bb3cfaeecaab315) preregistration; data and stimuli available at https://osf.io/w3cez/?view_only=2a45b9e2777143baa62f5a9f8c33e201.

3. We initially conducted factor analysis, finding that competence loaded onto the warmth dimension. Given research by Fiske et al. (2006), we left competence separate from warmth, though results were the same with competence included in the warmth composite. Moreover, competence may be distinct from power because it relates more to prestige, whereas power relates more to dominance (Cheng et al., 2013).

4. These correlations had 159 degrees of freedom.
